# Electroactive ecosystem insights from corrosion microbiomes inform gut microbiome modulation

**DOI:** 10.1093/ismejo/wraf112

**Published:** 2025-05-31

**Authors:** Liam M Jones, Sahar El Aidy

**Affiliations:** Department of Microbiome Engineering, Swammerdam Institute for Life Sciences, University of Amsterdam, Science Park 904, 1098 XH Amsterdam, Amsterdam, The Netherlands; Department of Microbiome Engineering, Swammerdam Institute for Life Sciences, University of Amsterdam, Science Park 904, 1098 XH Amsterdam, Amsterdam, The Netherlands; Amsterdam Microbiome Expert Centre, University of Amsterdam, Science Park 904, 1098 XH Amsterdam, Amsterdam, The Netherlands

**Keywords:** biofilms, extracellular electron transfer, electromicrobiome, electroactive microorganisms, gut microbiome, microbiologically influenced corrosion

## Abstract

Electroactive microorganisms influence environmental and host-associated ecosystems through their ability to mediate extracellular electron transfer. This review explores parallels between electroactive microorganisms (EAM)-driven microbiologically influenced corrosion systems and the human gut microbiome. In corrosion, EAMs contribute to biofilm formation, redox cycling, and material degradation through mechanisms such as direct electron transfer and syntrophic interactions. Similarly, gut-associated EAMs regulate redox balance, drive short-chain fatty acid production, and shape host–microbe interactions. Despite differing contexts, both systems share traits like anoxic niches, biofilm formation, and metabolic adaptability. Insights from well-characterized corrosion microbiomes offer valuable frameworks to understand microbial resilience, electron transfer strategies, and interspecies cooperation in the gut. Bridging knowledge between these systems can inform microbiome engineering approaches aimed at promoting gut health, highlighting the need for further functional metagenomics and exploration of archaeal contributions to biofilm stability and redox modulation.

## Introduction

The study of microorganisms capable of extracellular electron transfer (EET), known as electromicrobiology (Box 1), has emerged as a dynamic field within bioelectrochemistry and microbiology. Central to this discipline are bacteria and archaea, which can facilitate EET by transferring electrons across their cell membranes to external surfaces. Electroactive microorganisms (EAM) are traditionally defined by their ability to exchange electrons with electrodes, but they also thrive in many electrode-free systems, interacting with other microbial species, minerals, or soluble electron acceptors and donors [1].

Box 1 Key terminology.

Electroactive microorganisms (EAM): Microorganisms capable of transferring electrons to/from external surfaces, active in environments like corrosion systems and the gut microbiome.

Extracellular electron transfer (EET): Microbial process of transferring electrons to/from extracellular donors or acceptors, essential for energy production.

Direct electron transfer (DET): Electron transfer directly from the microorganism to an external acceptor via membrane-bound cytochromes or nanowires.

Mediated electron transfer (MET): Electron transfer facilitated by soluble mediators (e.g. flavins, quinones) between the microorganism and external acceptor.

Direct interspecies electron transfer (DIET): Electron exchange directly between microbial species without soluble shuttles, enabling syntrophic metabolism.

Terminal electron acceptor (TEA): Final electron acceptor in microbial respiration (e.g. metals, sulfate, nitrate, CO₂), critical for completing redox cycles.

Respiratory EAM: EAMs that respire using solid-phase electron acceptors (e.g. metals, electrodes), coupled to energy generation.

Fermentative EAM: EAMs using fermentation as primary metabolism and EET to maintain redox balance, often via soluble shuttles.

Microbiologically influenced corrosion (MIC): Metal corrosion driven by microbial activity, often involving EAM-mediated electron transfer.

Electrical MIC (EMIC): Corrosion via microbial EET, altering metal oxidation states electrochemically.

Chemical MIC (CMIC): Corrosion driven by microbial metabolites creating corrosive conditions.

Short-chain fatty acids (SCFAs): Organic acids (e.g. acetate, propionate, butyrate) produced by gut microorganisms; key for gut health and energy.

Syntrophic interactions: Microbial cooperation where one species’ metabolic products serve as substrates for another, vital in anoxic environments.

**Table 1:** Comparison of EAM in corrosion and gut microbiomes.

	Corrosion microbiome	Gut microbiome	Benefit of comparative study
Key microbial players	*Geobacter*, *Shewanella*, and *Desulfovibrio* spp. are well characterized for their role in MIC.	*Enterococcus*, *Faecalibacterium*, *Clostridium*, spp. are closely associated with overall gut health. Whilst species such as *Listeria*, *Klebsiella*, and *Desulfovibrio* spp. are linked to microbiome imbalance.	Identifies shared EET mechanisms across environments.
EET mechanisms	DET, MET, DIET	DET, MET	Reveals conserved and environment-specific electron transfer strategies.
Primary electron acceptors	Fe^3+^, SO₄^2−^, NO₃^−^	Fe^3+^, flavins, quinones	Helps understand microbial respiration under anaerobic conditions.
Biofilm phenotype	Enhances corrosion via MIC, biofilm mineralization	Promotes redox balance, mucosal protection	Provides insights into biofilm resilience and disruption strategies.
Impact on system/host	Causes metal degradation and infrastructure damage	Modulates gut redox homeostasis and immunity	Understanding EAM control can aid in MIC prevention and gut health therapies.
Redox interactions	Drives corrosion, supports syntrophic communities	Supports microbial cross-feeding and gut homeostasis	Enhances knowledge of redox balance in microbial ecosystems.
Industrial/medical implications	Pipeline protection, bioremediation [101], microbial fuel cells	Probiotic development, gut disease modulation	Cross-application of bioengineering strategies for beneficial outcomes.

EAMs employ two primary metabolic strategies: respiratory and fermentative. Respiratory EAMs couple EET with energy conservation by using solid-phase electron donors like metals or electrodes, often relying on direct electron transfer (DET) via outer membrane cytochromes or conductive nanowires. This process is critical to energy metabolism in environments like sediments and corrosion systems, as these environments often lack soluble electron acceptors. In contrast, fermentative EAMs primarily generate energy through substrate-level phosphorylation and utilize EET as a secondary pathway, often through mediated electron transfer (MET) using soluble electron shuttles such as flavins and quinones. These processes have been widely studied in various environments, including marine sediments, industrial equipment, and corrosion systems, where EAM contribute to biogeochemical cycles and material degradation [2].

Corrosion systems are driven by EAM, such as *Shewanella* and *Desulfovibrio* spp., which facilitate microbiologically influenced corrosion (MIC) through DET with metallic surfaces or by modifying the local electrochemical environment [3] (Table 1). Biofilm formation by diverse microbial communities, including EAM, enhances electron exchange, leading to material degradation [4]. In these anoxic environments, respiratory EET is a highly efficient energy-generating mechanism, allowing EAM to outcompete other microorganisms by utilizing metals as electron donors or acceptors [5–7].

The human gastrointestinal (GI) tract, which is home to a complex ecosystem of bacteria, which contribute to digestion, metabolism, and immune function [8–10], also harbors EAM, where their role is more auxiliary rather than relying on metals. Unlike corrosion systems, where EET drives metal oxidation and reduction, gut-associated EAM rely on MET to maintain redox balance in the anoxic environment [1, 11], supporting microbial interactions and regulating metabolic outputs such as short-chain fatty acids (SCFAs) critical for host health. EAM are implicated in regulating gut health and affecting inflammatory processes associated with gut microbiota. These bacteria interact, directly or indirectly, with epithelial enterocyte cells in the gut through electron exchange, affecting gut homeostasis and the broader metabolic network [12] (Table 1). *Desulfovibrio* spp. which are also present in the gut, influence microbial fermentation dynamics through their utilization of hydrogen gas, but their role is secondary to fermentative metabolism. Similarly, *Faecalibacterium prausnitzii* has been shown to perform EET using flavins as mediators [13], this function appears to be more related to oxidative stress resistance than to a dominant role in gut metabolism.

This review explores the parallels in the roles and mechanisms of EAM in corrosion and gut microbiomes, two environments where EET plays a crucial yet context-dependent role. Whereas much is known about the evolution, genetics, and mechanisms of EET in corrosion systems, revealing insights into microbial adaptation, electron transfer mechanisms, and biofilm dynamic, the role of EET in the gut microbiome remains poorly understood [1, 2, 14, 15]. Key questions include how gut-associated EAMs regulate EET under dynamic redox conditions and the extent to which these processes influence host physiology, immune responses, and pathogen exclusion. By learning from the well-characterized corrosion systems, we aim to uncover fundamental ecological and colonization principles that could inform our understanding of EET in the gut microbiome. Comparative studies on microbial adaptation strategies, electron transfer mechanisms, redox homeostasis, and biofilm dynamics, may provide valuable insights into microbial interactions in the gut, potentially guiding interventions like engineered probiotics or dietary modulation of redox-active compounds. Whereas MIC mitigation strategies involve quorum sensing (QS) inhibitors and biofilm-disrupting agents, similar principles could be explored for gut microbiome modulation. Conversely, microbial strategies from the gut, such as redox balancing mechanisms, may also hold potential for corrosion mitigation, though this is beyond the scope of this review. Ultimately, this review aims to translate knowledge from corrosion microbiomes to better understand and potentially manipulate redox-sensitive microbial interactions in the gut.

## Translating survival strategies from corrosion environments to the gut microbiome

Both corrosion systems and the human gut microbiome share several key environmental traits that support the growth of EAM. Their ability to form biofilms, adapt to fluctuating conditions, thrive in anoxic environments, and exhibit remarkable metabolic adaptability are among the most striking similarities between these systems. These similarities, which we discuss herein, underline the adaptive strategies of EAM, which allow them to flourish across a wide range of ecological niches.

### Biofilm phenotype

Among the survival strategies shared by corrosion systems and the gut microbiome, biofilm formation stands out as a central mechanism. This adaptive strategy (Box 2) is widely employed by microorganisms across natural, industrial, and host-associated environments. In both corrosion and gut microbiomes, biofilms enhance microbial persistence, community stability, and interaction with surfaces or host tissues. Quorum sensing (QS), a form of cell-to-cell communication, plays a key role in the regulation of biofilm formation, where microorganisms coordinate gene expression based on cell density [16]. This enables efficient production of extracellular polymeric substances (EPS) and facilitates the development of complex, stable biofilm architectures [17–19].

Box 2 What are biofilms?

Biofilms are structured communities of microorganisms embedded within a self-produced matrix of extracellular polymeric substances (EPS), which typically include proteins, polysaccharides, and nucleic acids [17–19, 98, 99]. Some biofilms may lack or minimize EPS production and instead rely on other strategies. For example, some gut biofilms, such as those formed by *Akkermansia muciniphila*, integrate into host-derived mucus rather than producing their own EPS [73]. EPS production is dynamic and it depends on social cooperation through quorum sensing (QS). QS controls EPS production, structural organization, and dispersion, affecting biofilm resilience in diverse [100].

**Key steps in biofilm formation:**

1) Initial adhesion: Planktonic (free-floating) microorganisms reversibly attach to a surface via weak forces (e.g. van der Waals forces or pili interactions).
2) Irreversible attachment: Cells strengthen adhesion to the surface by producing EPS, which also helps protect them from external stressors.
3) Irreversible attachment: Cells strengthen adhesion to the surface by producing EPS, which also helps protect them from external stressors.
4) Maturation: As biofilms mature, they develop complex 3D architectures, with nutrient gradients and spatially distinct metabolic zones. These structures enhance microbial resilience.
5) Dispersion: Environmental cues or nutrient limitations cause biofilm cells to detach and colonize new surfaces, ensuring biofilm persistence in diverse habitats.

**Adaptation strategies in biofilms:**

- Nutrient utilization: Mixed-species biofilms enable co-metabolism, where different species exchange nutrients and enhance resource use.
- Horizontal gene transfer: Biofilms are hotbeds for horizontal gene transfer, which increases microbial diversity and allows for rapid adaptation to environmental changes.
- Cooperation and competition: Biofilms are sites of both cooperative (e.g. syntrophic interactions) and competitive (e.g. resource competition) behaviors that optimize survival.
- Resistance to Stressors: Biofilms confer protection against biocides, antibiotics, and extreme environmental conditions, making microorganisms more resilient.

#### Biofilms in corrosion systems

In corrosion environments, biofilms typically develop on metal surfaces, where they initiate and accelerate MIC. Whereas biofilm formation generally begins with planktonic cells attaching to a surface and transitioning to sessile cells that produce EPS (Box 2), this process in corrosion environments involves unique microbial interactions that lead to corrosion. Specifically, biofilms in corrosion systems can promote both electrical microbial influenced corrosion (EMIC) and chemical microbial influenced corrosion (CMIC) [20]. MIC is not caused by any one microorganism, but is associated with mixed-species microbial communities.

EMIC is primarily electrochemical, driven by microbial EET, where electroactive bacteria transfer electrons to metal surfaces, influencing metal oxidation and reduction. Key players in this process include EAM, such as *Geobacter* [21] and *Shewanella* [22, 23] spp. which use outer membrane cytochromes and conductive nanowires to facilitate electron exchange with metal surfaces. Some EAM, like *Shewanella* spp. secrete soluble redox-active flavins that shuttle electrons between the biofilm and the metal surface, thereby influencing corrosion [24].

CMIC is driven by the production of metabolites, such as acids, which create a corrosive microenvironment. For example, sulfate-reducing bacteria (SRB) such as *Desulfovibrio* sp. [25] produce hydrogen sulfide which reacts with metal surfaces to form iron sulfide, which accelerates localized corrosion. SRB can withdraw electrons directly from the metal surface through membrane-bound redox proteins via DET or indirectly through redox mediators via MET [26]. CMIC can also occur via microbial actions that generate differential aeration cells or through enzymatic degradation of the surface material [4, 5]. Both EMIC and CMIC often coexist, with biofilms using chemical and electrical processes synergistically to accelerate corrosion.

Archaea have demonstrated potential EET capabilities via direct interspecies electron transfer (DIET) [27]. DIET is a syntrophic metabolic process where electrons are directly exchanged between microorganisms, bypassing soluble electron shuttles [1]. *Methanosarcina barkeri* can directly capture electrons from coexisting microbial species, such as *Geobacter metallireducens* [28], within a biofilm community [29]. Here, spatial organization within biofilms plays a pivotal role in facilitating cooperative metabolism. It was previously demonstrated that in *Geobacter* communities, conductive pili mediate DIET, enabling syntrophic cooperation between distinct species via physical electron conduction pathways [30]. Conductive pili in *Geobacter* spp. not only conduct electrons but also contribute to structural integrity and cellular aggregation within the biofilm matrix. These processes alter the kinetics of corrosion reactions [31], causing significant damage in industries such as energy [32], water systems [33], and marine environments [34].

Biofilms in corrosion environments confer protection to microorganisms from harsh chemical treatments, such as biocides that are commonly used to combat MIC [35]. This protection, combined with the cooperative syntrophic interactions and horizontal gene transfer within biofilm communities, enhances microbial survival and resilience in corrosive environments. EAM within these biofilms adapt to oxidative stress and other harsh conditions, thereby contributing to the persistence and spread of corrosion. MIC-associated EAM alter the physical and chemical properties of surface materials, changing their susceptibility to corrosion [36].

Overall, biofilm formation and microbial EET play vital roles in the progression of MIC, with mixed-species biofilms often enhancing the rate of metal degradation through synergistic electrical and chemical processes. Understanding these mechanisms in corrosion biofilms provides insight into how similar strategies may apply to other environments, such as the gut microbiome, where microbial communities similarly form biofilms to maintain stability and contribute to host health.

#### Gastrointestinal tract biofilms

Biofilm formation also occurs within the lumen and the mucus layer covering the intestinal epithelium in the GI tract, promoting steady, long-term colonization while allowing bacteria to stay near their food sources [37]. Understanding how GI biofilms are spatially organized, including microbial stratification, heterogeneity, and localization along the tract, is key to grasping microbiome function and its impact on host health [38, 39]. The gut microbiome is spatially organized based on nutrient availability, oxygen gradients, and host factors, creating distinct environmental niches in both the lumen and mucus [40]. Biofilms in the gut form multi-layered structures where microorganisms engage in syntrophic relationships, with primary fermenters supplying substrates to secondary degraders [41]. This spatial organization makes EET crucial for maintaining redox balance, and co-metabolism, as bacteria share metabolic intermediates and distribute redox burdens [38, 39]. For example, *Enterococcus faecalis*, Ebp pili are implicated in both adhesion to host tissues and EET, suggesting that multifunctional pili may be a conserved feature supporting both biofilm architecture and redox interactions across diverse ecosystems [42]. By coupling EET with fermentation, gut bacteria can regenerate intracellular NAD+, and extracellular redox shuttles such as flavins, quinones, and phenazines more efficiently, which leads to enhanced SCFA production, which benefits host metabolism [43]. Intracellular electron shuttles primarily operate inside the cell to drive redox reactions in metabolic pathways, while extracellular electron shuttles act as mediators, transferring electrons between microorganisms or to solid-phase acceptors [44]. EET-active bacteria can also modulate iron availability in the gut, influencing microbial nutrient acquisition and pathogen competition [45] and help regulate oxidative stress by maintaining redox homeostasis in the gut mucosa [46, 47].

Distinct regions of the GI tract harbor microbial communities that influence metabolism, immune interactions, and disease susceptibility. To sustain anoxic microenvironments that support electron transfer processes such as fermentation and anaerobic respiration, biofilm production is crucial for EAM in the gut. *Bacteroides* sp. [48, 49], for example, form biofilms that aid in the breakdown of complex polysaccharides, which are subsequently fermented by Bacillota like *Ruminococcus* sp., leading to SCFAs production that benefits the host [50]. The biofilm matrix also promotes syntrophic interactions, where various bacterial species exchange electrons, impacting gut health and microbial community dynamics [37, 51]. For instance, mucosa-associated methanogens, capable of biofilm formation [52], such as *Methanobrevibacter smithii*, rely on hydrogen produced by fermentative bacteria like *Clostridia* for methanogenesis, maintaining low hydrogen partial pressure that favors continued fermentation by primary producers [53]. Extracellular DNA (eDNA) and mucins in the gut mucus layer may enhance EET by serving as conductive scaffolds or mediators that facilitate electron flow between microbial cells and terminal electron acceptors, potentially influencing microbial metabolism and community dynamics [54].

Many commensal and pathogenic gut inhabitants have genes for EET and/or demonstrate electroactivity. For example, *Clostridium cochlearium* and *Limosilactobacillus reuteri* [55]*,* both capable of forming biofilms [56, 57], exhibit electroactivity [56, 58]. These species support gut health by maintaining microbial balance, regulating immune responses, and contributing to metabolic functions. *C. cochlearium* leverages EET mechanisms, such as MET using unknown redox shuttles, to thrive under low-oxygen conditions [56]. *Lactobacillus reuteri* shuttles electrons via heme-containing cytochromes and menaquinones. This electron transfer promotes gut health by producing lactic acid, which lowers intestinal pH, thereby creating an environment less hospitable to pathogenic microorganisms. Here, EET may be associated with lactic acid production, as electron transfer helps regenerate NAD^+^, sustaining glycolysis and acidogenesis. Together with other commensal species, these bacteria play a key role in fermentation, producing metabolites that serve as energy sources for the host. Conversely, some strains, such as *Desulfovibrio desulfuricans* [59], can produce inorganic compounds like hydrogen sulfide, contributing to sulfur cycling, with both positive and negative effects [9]. Whereas low concentrations of hydrogen sulfide may support epithelial cell health, elevated levels can be toxic, disrupting barrier function and contributing to diseases like inflammatory bowel disease (IBD) and colorectal cancer (CRC) [60, 61].

#### What corrosion biofilms teach us about the gut microbiome

Whereas biofilm structures in the gut and corrosion systems form in distinct environments, they share fundamental principles of microbial interactions and community stability. Yet, since corrosion biofilms are better studied, they offer valuable insights for understanding and manipulating gut biofilms. In corrosion systems, EET plays a key role in microbial communication and metabolic stability. Similar processes may also be crucial in the gut for maintaining redox balance and metabolic function. Techniques from corrosion research, such as electrochemical mapping, could enhance our ability to measure and manipulate EET in gut biofilms, offering new strategies for microbiome modulation. The extracellular matrix in corrosion biofilms is essential for structural integrity, facilitating microbial interactions and nutrient exchange. Insights into matrix-driven microbial organization in corrosion systems could inform studies of gut biofilm architecture, including nutrient flow, spatial structuring, and community resilience. Corrosion biofilms exhibit resilience to environmental stressors like metal toxicity and nutrient fluctuations. This resilience stems from both biofilm structure and microbial metabolic flexibility, an insight that could help explain how gut biofilms withstand digestive enzymes, pH shifts, and dietary changes. Corrosion biofilms highlight the role of EAM in maintaining biofilm stability and function. In the gut, microorganisms with specialized electroactive capabilities might similarly contribute to microbiome balance and health. Studies of surface interactions in corrosion biofilms, considering properties like roughness, hydrophobicity, and charge, may shed light on how gut bacteria engage with the epithelium, mucus, and dietary particles to shape microbial composition and function.

### Environmental adaptation

Corrosion and gut microbiomes are dynamic environments requiring unique adaptation mechanisms. Microorganisms in these environments must withstand fluctuating redox conditions, resource competition, and interaction with surfaces, whether host tissue or material surfaces. Corrosion biofilms, often dominated by SRB and iron reducing bacteria (IRB) [62], utilize extracellular matrices and redox-active compounds to modulate their environment; a strategy mirrored in gut biofilms by electroactive and mucosa-associated bacteria. These shared mechanisms highlight universal microbial survival strategies, offering insights into how microbial communities persist and shape their environments despite their vastly distinct ecological contexts.

#### Corrosion system environments

In corrosion systems, EAM encounter harsh chemical conditions, including fluctuations in pH, limited nutrients [63], high salinity, and toxic compounds such as heavy metals. These chemical conditions vary significantly among corrosion systems. For example, pH fluctuations can range from acidic conditions (pH < 5) to more alkaline conditions (pH > 9). Acidic conditions may arise abiotically via thermochemical sulfate reduction producing sour gas (hydrogen sulfide), or biotically as a direct result of microbial acid production [64]. Similarly, alkaline conditions can be abiotic or biotic, resulting in sweet gas (carbon dioxide) formation [64]. Salinity and water chemistry are also highly variable. Seawater environments often have high salinity, while some oil and gas reservoirs contain high levels of calcium, magnesium, sulfate, and bicarbonate. These factors significantly influence corrosion processes [64]. The presence of toxic compounds such as heavy metals (Fe, Mn, Ni, Zn, Pb, Cr, As) and hydrocarbons can shape microbial communities, and, in some cases, exacerbate MIC. Physical and redox conditions, such as oxygen gradients, temperature, and pressure, can also be highly variable across different corrosion systems [64]. The combination of extreme pH, salinity, toxic metals, and redox gradients creates a highly stressful environment demanding adaptive strategies from biofilm communities. Microbial survival mechanisms include the use of alternative electron acceptors, metal resistance systems, and the formation of protective EPS in biofilms [65]. Microbial adaptation to these multifaceted stressors is essential for survival and includes expression of metal resistance genes, production of conductive nanowires and redox-active proteins, and biofilm formation (see section above). Generally, these adaptation strategies enhance resilience and drive localized corrosion. The biofilm phenotype also facilitates the retention of electron shuttles, metal ions, and redox gradients, establishing microenvironments that promote metal dissolution and pitting corrosion. Direct microbial interaction with metal surfaces, either through cytochrome-mediated DET or nanowire networks, creates continuous electron flow from the metal (acting as an anode) to microbial electron acceptors, directly linking microbial metabolism to corrosion processes.

Several key EAM species have been identified in corrosion environments, with distinct mechanisms for electron transfer and metal interaction. Among the most prominent are *Desulfovibrio vulgaris*, *Geobacter sulfurreducens*, and *Shewanella oneidensis*. These are classic contributors to MIC. Altogether, these corrosion-associated EAMs not only adapt to extreme conditions but actively modulate redox dynamics, influencing both the rate and nature of material degradation in corrosion systems. Microbial mechanisms of EET were introduced earlier and will be discussed in more detail in a subsequent section.

#### Gastrointestinal tract environments

The GI tract is highly dynamic and exhibits gradients in nutrient availability, pH, and oxygen which vary based on biogeography. The stomach is acidic and oxygen-rich, but as food moves into the small intestine, conditions change. The presence of bile acids [49], digestive enzymes, and antimicrobial peptides, creates a selective environment that shapes microbial composition [37]. Mucus thickness also varies along the GI tract, reflecting differences in function, microbial load, and exposure to luminal contents [66]. The mucus layer serves as a protective barrier, preventing direct microbial contact with epithelial cells [67] while also facilitating nutrient absorption and immune regulation. In the small intestine, mucus thickness ranges from approximately 100–500 μm across the duodenum, jejunum and ileum [68, 69]. The duodenum is relatively acidic and strongly oxic, but as food moves through the jejunum and ileum, pH increases and oxygen levels decline in [70, 71]. In the colon, mucus is thought to be organized into two distinct layers. The inner mucus layer (firmly attached to the epithelium) is low in oxygen. Its primary function is to serve as a physical barrier and it can have a thickness ranging from 100–300 μm [68, 69]. In contrast, the outer mucus layer is looser and more exposed to the lumen which has less oxygen compared to the inner mucus. This allows for strict anaerobes to thrive, with species such as *Akkermansia muciniphila*, which has been implicated in using pili-like proteins in immune modulation [72, 73]. This outer mucus has a thickness ranging from 400–700 μm. Oxygen tension varies along the longitudinal axis of the gut, with the highest luminal oxygen concentration in the duodenum and the lowest in the caecum and colon. Thus, colonic epithelial cells live in chronic hypoxia [74]. Mucus thickness also increases from proximal to distal colon, providing stronger protection against fecal material and microbial fermentation by-products [68, 69]. The colon itself is anoxic, nutrient-rich and contains fermentable fibers and supports diverse microbial metabolic activities, including high SCFAs production [37]. Among the EAM found in the gut, *Bacteroides fragilis* [75] and *C. cochlearium* [56], have adapted to fluctuating oxygen levels, particularly during the transition from the small to the large intestine, in response to increasing anaerobic conditions. *B. fragilis* utilizes diverse electron acceptors and upregulates stress-response genes to cope with transient oxidative stress [75]. This adaptability not only ensures survival but also contributes to redox balance and microbial stability within the gut ecosystem.

#### Insights from corrosion environments for the gut

Corrosion environments present extreme conditions that require EAM to develop robust survival mechanisms. For example, *Shewanella* and *Geobacter* spp. can modify their metabolic pathways to utilize available inorganic electron acceptors, enabling persistence under stress. These bacteria often possess metal efflux pumps, detoxifying systems, and specialized metabolic pathways, adaptations that mirror the stress responses seen in the dynamic environment of the gut microbiome. By studying how MIC-related microorganisms adapt to these harsh conditions, we can gain insights into how gut microorganisms navigate fluctuating conditions, including how pathogens evade gut defenses and how beneficial microorganisms persist, sometimes even thriving under stress. This understanding may inspire novel therapeutic strategies, such as modulating gut redox states to influence microbial community dynamics and improve human health.

### Metabolic versatility

The shared metabolic versatility and electron transfer capabilities of EAM in corrosion and gut environments highlight evolutionary strategies that enable microbial survival in fluctuating conditions. In both systems, EAM display remarkable metabolic versatility, allowing them to exploit a wide range of electron donors and acceptors to sustain metabolism under dynamic environmental pressures. This adaptability supports microbial survival in environments where nutrient and energy sources vary dynamically. Exploring these parallels not only enhances our fundamental understanding of microbial ecology but also opens avenues for innovative biotechnological applications in health and industry.

**Figure 1:**
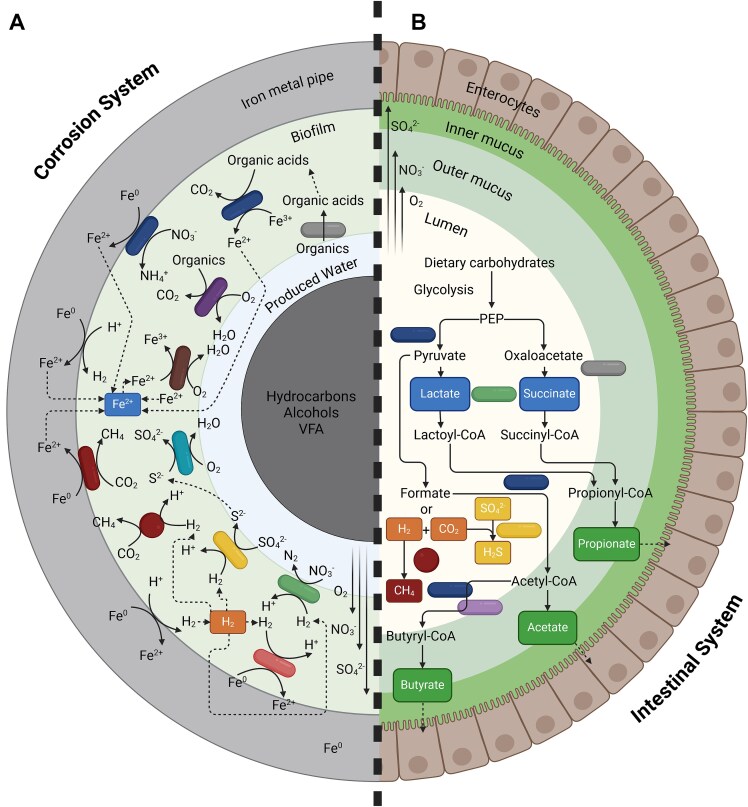
(A) Key microbial pathways involved in MIC of ferrous metals. Hydrogen gas (H₂) released from metallic iron (Fe^0^) via the HER and produced by fermentative bacteria is consumed by denitrifiers, sulfate reducers, methanogens, and acetogens. Heterotrophs, sulfide oxidizers, and iron oxidizers consume O₂, promoting corrosion under anaerobic conditions. Electroactive methanogens and Fe^3+^ reducers extract electrons directly from Fe^0^, producing Fe^2+^, which iron oxidizers convert back to Fe^3+^ oxides, serving as electron acceptors. Diffusion of organics and electron acceptors (O₂, NO₃^−^, SO₄^2−^) into the biofilm, combined with Fe^0^ availability, creates vertical heterogeneity of functional populations. Adapted from [5]. (B) Key microbial pathways in the gut microbiome leading to major metabolites from carbohydrate fermentation and bacterial cross-feeding. SCFAs include acetate, produced via acetyl-CoA or the Wood–Ljungdahl pathway (acetogens), butyrate (from acetyl-CoA by Bacillota), and propionate (by Bacteroidota from carbohydrates, or Bacillota from lactate/succinate). Adapted from [9, 10].

Microbial communities in both electromicrobiomes function as interconnected bioenergetic networks, where EET and other cooperative pathways support metabolic stability and adaptation [43]. Electroactivity enables the formation of metabolic food webs, where respiratory and fermentative electroactive microorganisms cooperate to oxidize organic compounds, supporting co-metabolism, cross-feeding, and syntrophic interactions within microbial communities. These complex functional roles of electroactive microorganisms in both corrosion and gut systems are reflected in the organization of electron flow and interspecies interactions (Fig. 1). In corrosion environments, EAM recycle metals and sulfur compounds [76], sustaining microbial respiration and promoting resilience against environmental stress. In the gut, SCFAs, cofactors such as B vitamins, heme, quinones, and electron carriers help optimize energy flow across the microbial community and allow for collective survival in challenging or nutrient-scarce conditions [41, 77]. The ability of EAM to recycle nutrients and sustain cooperative metabolic processes, exemplifies how metabolic versatility drives microbial resilience in complex environments. Whether in corrosion systems, where they influence material degradation, or in the gut, where they support host–microbe interactions, these microorganisms play crucial roles in ecosystem function [43].

**Figure 2:**
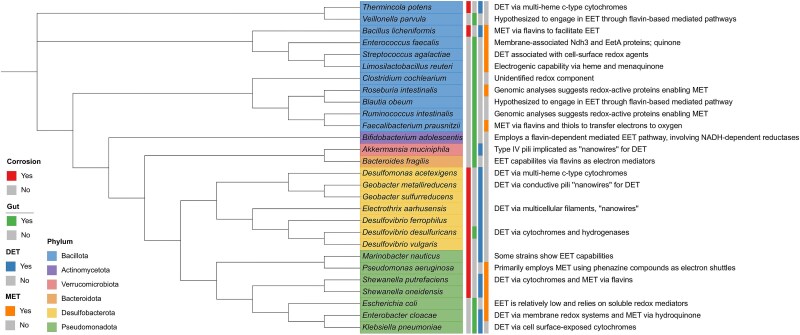
Phylogenetic diversity of MIC- and gut-associated microorganisms. Phylogenetic diversity of select MIC-associated and gut-associated microorganisms based on published whole- genome or metagenome sequences. The bacterial species shown are not exhaustive and does not include archaea. MIC-associated microorganisms were selected based on documented EET capabilities in the literature. Gut-associated microorganisms selected have either been characterized for their EET capabilities in the literature or have an unknown mechanism. DET mechanisms and MET mechanisms are shown with a description of the mechanisms for each species. The phylogenetic tree was created using interactive tree of life (iTOL).

#### Metabolic networks in corrosion systems

Redox gradients, which occur in biofilms formed on metal surfaces or due to the natural properties of the environment, such as in deep marine sediments or oil and gas pipelines, largely influence metabolic dynamics in corrosion systems. On metal surfaces, an oxygenated outer layer coexists with a deep anoxic zone and differential aeration cells that drive key processes such as sulfate reduction, and metal oxidation. These conditions create microenvironments where EAM thrive by leveraging diverse electron acceptors to sustain metabolic activity. For example, *Geobacter* sp. directly transfers electrons to metal surfaces via DET, a process that is central to EMIC [78]. Key metabolic reaction pathways associated with MIC of ferrous metals involve diverse microbial communities engaging in redox cycling and EET-dependent interactions, including hydrogen metabolism and metal reduction (Fig. 1A) [5]. A critical aspect of corrosion-related microbial metabolism is the cycling of both hydrogen gas and ferrous iron. Hydrogen gas, produced via the hydrogen evolution reaction (HER) and by fermentative microbial activity, serves as an electron donor for denitrifiers, sulfate reducers, methanogens and acetogens. Each microbial group performs distinct redox reactions that contribute to corrosion processes. Similarly, ferrous iron produced via the HER and by IRB is subsequently utilized by iron oxidizing bacteria [5], thus sustaining a dynamic redox balance. Together, these syntrophic interactions enhance redox cycling, cross feeding, and biofilm stability and exacerbate corrosion. Aerobic heterotrophs, sulfide oxidizing bacteria, and iron oxidizers consume oxygen near the biofilm surface, promoting anaerobic conditions favorable for corrosion. The diffusion of electron acceptors like oxygen, nitrate [79], and sulfate into the biofilm, combined with the availability of Fe^0^ at the base, creates a stratified microbial community exhibiting high metabolic versatility and interdependence, driving continuous corrosion.

EAM exhibit remarkable metabolic flexibility, where they can utilize organic compounds, hydrogen, sulfides, nitrates, and even reduced metal ions as electron donors [5]. For example, *Shewanella* sp. can oxidize lactate, formate, or hydrogen, while reducing a variety of electron acceptors, including ferric iron and manganese oxides [80]. Organic compounds present in corrosion environments, originating from industrial pollutants, hydrocarbons, or even biofilms, further support microbial metabolic diversity. This metabolic versatility enables microbial survival in dynamic conditions, where fluctuations in oxygen levels, metal ion availability, and redox gradients shape microbial interactions.

#### Gastrointestinal tract nutrient dynamics

In the gut microbiome, EAM exhibit a similar breadth of metabolic capabilities, fermenting dietary polysaccharides, proteins, peptides and mucins, degrading amino acids, and participating in sulfur and nitrogen cycling [9, 10]. For example, *Desulfovibrio* spp., common MIC-associated microorganisms, also reduce sulfate to hydrogen sulfide in the gut microbiome [60]. *E. coli* [81]*,* which ordinarily ferment sugars into organic acids and gases or metabolize amino acids, can shift to nitrate as an electron acceptor, reducing nitrate to ammonia, thus gaining a competitive advantage in inflamed gut environments [82]. Key metabolic pathways in the gut microbiome center on carbohydrate fermentation and microbial cross-feeding, where metabolites such as SCFAs are produced and exchanged among taxa with distinct metabolic roles (Fig. 1B) [9, 10].Various bacteria metabolize carbohydrates into key metabolites. Acetate is produced broadly by enteric bacteria via pyruvate metabolism and by acetogens through the Wood–Ljungdahl pathway. Butyrate is formed from acetyl-CoA by several Bacillota families, while propionate is synthesized by Bacteroidota from carbohydrates and by some Bacillota through lactate or succinate pathways. Hydrogen gas is a central intermediate, generated during fermentation and consumed by methanogens and acetogens to maintain redox balance. Cross-feeding interactions are prominent, with certain bacteria converting fermentation intermediates like lactate and succinate into SCFAs. This metabolic versatility and interdependence contribute to a stable, efficient microbial ecosystem optimized for energy extraction and nutrient recycling within the gut environment.

Just as redox gradients drive metabolic interactions in corrosion systems, the gut lumen features a steep oxygen gradient, with the mucosal layer being microaerophilic or anoxic (see section *Gastrointestinal Tract Environments*, described above). This supports fermentation and anaerobic respiration, where electron transfer mechanisms influence microbial dynamics. For example, *E. faecalis* utilizes flavins to shuttle electrons to extracellular electron acceptors, thereby enhancing its metabolic activity [83]. Similarly, syntrophic interactions regulate hydrogen cycling; fermentative bacteria generate hydrogen, which is consumed by sulfate reducers (*D. desulfuricans*), acetogens (*Blautia* obeum) [84], and methanogens (*M. smithii*). This competition highlights a metabolic interdependence, where acetogens and methanogens prevent hydrogen accumulation, ensuring that fermentative bacteria can maintain their energy production. At the same time, *F. prausnitzii* utilizes acetate to synthesize butyrate, a key metabolite for gut health and serves as an energy source for host cells [85]. SRB and methanogens further influence the sulfur and carbon cycles in the gut, respectively, establishing niches that sustain microbial diversity. The interactions among these microorganisms underscore the complexities of gut metabolism, where microbial EET mechanisms and metabolic activities are intricately linked to maintaining a stable and functional gut environment.

#### Advancing microbial metabolism in the gut through insights from corrosion microbiomes

The metabolic flexibility of EAM in corrosion systems highlights their ability to switch between electron donors and acceptors based on availability. A similar metabolic adaptability in the gut allows microorganisms to adjust to fluctuations in nutrient availability driven by host diet and metabolism. In corrosion systems, MIC-associated EAM often engage in syntrophic relationships, where metabolic products are exchanged to optimize energy extraction. Understanding these cooperative interactions in corrosion systems can shed light on similar syntrophic processes in the gut, where electron transfer between microbial partners influences nutrient breakdown, fermentation, and overall gut health. This knowledge may lead to strategies to modulate microbial interactions in the gut, potentially impacting conditions associated with microbiome community imbalance.

### Microbial mechanisms of extracellular electron transfer

**Figure 3:**
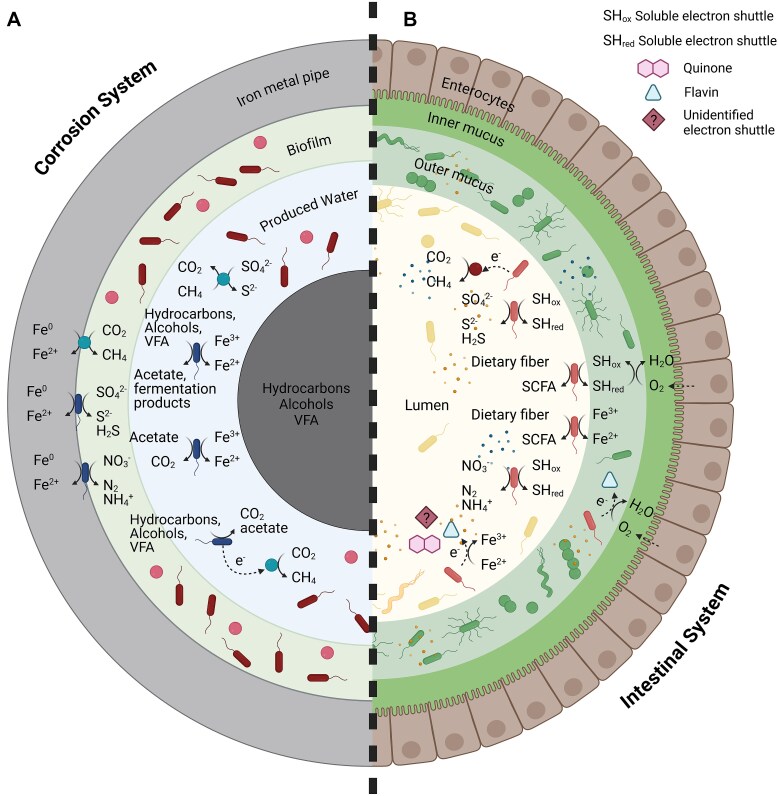
Functional roles of electroactive microorganisms, capable of extracellular electron transport, in different electromicrobiomes. Corrosion microbiome (A), and gut microbiome (B). Food chains made up of respiratory and fermentative electroactive microorganisms that work together to oxidize organic substances and permit co-metabolism and cross-feeding are made possible by electroactivity. In situations where Fe(III) is unavailable, direct interspecies electron transfer between syntrophic electron-donating bacteria and electron-accepting methanogens is crucial. The oxidized form of soluble electron shuttles (SH_ox_), which take up electrons from fermentative electroactive bacteria, are alternative electron acceptors in the gut. With O_2_ reduction, the shuttles’ reduced form (SH_red_) is transformed back into its oxidized form. In order to facilitate anaerobic respiration, electroactive corrosive microorganisms remove electrons from the metallic iron (Fe^0^) in structural materials either directly or mediated via electron shuttles.

Despite the distinct environmental conditions in corrosion and gut ecosystems, microbial species in both niches exhibit shared strategies for EET. Variations in physical, chemical, and biological factors shape the diversity of EAM in each system. Representative MIC- and gut-associated electroactive microorganisms span diverse phylogenetic lineages, reflecting both their evolutionary breadth and convergence in metabolic strategies relevant to extracellular electron transfer (Fig. 2). Species included in the phylogenetic analysis includes species with documented or inferred EET capabilities from the literature. Metadata such as source publications, genome accession numbers, and EET classification (DET, MET, or unknown) are provided in Supplementary material (Supplementary Table 1 and 2). These strategies underpin community-level interactions where respiratory and fermentative electroactive microorganisms participate in co-metabolism, syntrophy, and redox cycling, shaping functional food webs in both corrosion systems and the gut microbiome (Figs 1 and 3).

Across diverse bacterial lineages, microorganisms employ analogous EET mechanisms, including DET and MET. Whereas MIC-associated bacteria frequently utilize DET through outer membrane cytochromes and conductive nanowires, some gut-associated species exhibit homologous redox-active components, suggesting potential electron transfer capabilities that may influence host interactions or interspecies metabolism. This functional convergence underscores how environmental pressures drive microbial adaptation towards efficient electron transfer processes.

Key molecular components of EET include multi-heme c-type cytochromes [86], conductive pili (nanowires), and soluble electron shuttles such as heme molecules, flavones, and quinones. Well-characterized systems like the *omc* (outer membrane cytochrome) genes (OmcS, OmcZ) genes in *Geobacter* sp. [35], and *mtr* (metal-reducing) operon (MtrA, MtrB, MtrC, and MtrF) in *Shewanella* sp. [53, 81] are central to this process*.* These genes encode proteins that enable microorganisms to transfer electrons to or from insoluble electron acceptors/donors located outside the cell. The *mtr* operon encodes a series of proteins that form an electron conduit spanning the inner membrane, periplasm, and outer membrane, facilitating electron flow from intracellular metabolism to extracellular acceptors [87, 88]. The *omc* genes encode multiple outer membrane-associated multi-heme c-type cytochromes that form conductive filaments [89]. Other key proteins involved in EET include PpcA-E, a family of multi-heme c-type cytochromes essential for electron flow across the periplasm to outer membrane acceptors [90], and Ndh [2, 3], an NADH dehydrogenases supporting redox balance and electron transport [91]. NADH dehydrogenases such as Ndh2 and Ndh3 are implicated in maintaining intracellular redox balance in gut-associated bacteria, linking central metabolism to EET processes and influencing fermentation pathways and microbial competition [44]. In addition to these well-studied environmental electrogens, recent research highlights the presence and potential roles of EET-associated proteins within gut microbiota. For instance, PplA, a flavin-binding lipoprotein found in *Listeria Monocytogenes*, facilitates MET by acting as a soluble electron shuttle, supporting microbial colonization and energy conservation under anaerobic conditions [92]. Another notable protein, Amuc_1100, a pilin-like outer membrane protein expressed by *A. muciniphila*, contributes to electron transfer and also plays a key role in modulating host–microbe interactions, influencing gut barrier integrity and immune responses [93]. Understanding how these specialized EET mechanisms function in gut-associated EAMs sheds light on their ecological roles in shaping gut redox environments. These mechanisms impact host physiology and modulate gut microbial community dynamics. Further investigation into these mechanisms could provide valuable insights into their beneficial or detrimental effects within the gut ecosystem.

## Conclusions and outlook

Across both corrosion systems and the gut microbiome, EAM play a pivotal role in structuring their respective environments by mediating electron flow, which in turn drives broader community dynamics and ecosystem functions. Regardless of habitat, EAM influence local chemical gradients and redox conditions, creating microenvironments that support or destabilize structural and biological systems. In corrosion environments, microorganisms like *Desulfovibrio* spp. couple their metabolism to the oxidation of metal surfaces, utilizing metal ions as electron donors or acceptors. This electron transfer leads to reductive dissolution of protective metal oxides, localized acidification, and accumulation of corrosive by-products such as hydrogen sulfide, ultimately compromising the integrity of the metal substrate [94, 95]. Similarly, in the gut, EAM regulate electron flow through fermentation and redox cycling processes that structure microbial interactions and influence host physiology. Microorganisms like *F. prausnitzii* and *C. cochlearium* DET toward the production of SCFAs, fostering syntrophic relationships and stabilizing community function [13, 56].

In both environments, the control of electron flow has cascading effects. In corrosion systems, it alters the electrochemical landscape leading to material degradation, while in the gut, it shapes metabolic networks and nutrient availability, influencing host health. Certain species, whether *Desulfovibrio* spp. [59–61], in corrosion systems or in the gut, demonstrate how overactivity or shifts in redox balance can result in harmful outcomes, such as excessive hydrogen sulfide production leading to inflammation or structural breakdown. Opportunistic pathogens like *Klebsiella pneumoniae* [96] and pathogenic *E. coli* [81, 82] leverage changes in electron flow and nutrient dynamics to gain competitive advantage, disrupting system stability. Methanogens, present in both soil and gut environments, similarly play a conserved role by consuming excess hydrogen, maintaining favorable redox conditions, and facilitating continuous metabolic activity [97]. Whereas the specific outcomes differ, the underlying functional role of EAM is parallel. They mediate electron transfer processes that dictate the physical integrity of their environment and influence cooperative or competitive interactions within microbial communities. Their ability to control redox gradients makes them central players in both biofilm-driven corrosion and gut ecosystem balance.

Electromicrobiology research offers critical insights on microbial adaptation, survival tactics, and the variety of functions of these special microorganisms. Further metagenomic and functional analyses are essential to deepen our understanding, particularly of phylogenetic diversity and gene function in both MIC- and gut-associated microbiomes. A key knowledge gap lies in deciphering how biofilm-like communities in the gut, especially at the mucosal interface, stabilize and resist disturbances such as dietary shifts, antibiotic treatments, or inflammation. Although EPS and redox gradients are known to influence community resilience, the specific microbial communication strategies governing this stability remain unclear. Electron sinks, including hydrogen, nitrate, and sulfate, play critical roles that warrant further investigation in the gut. Drawing parallels from corrosion systems, where electron acceptor availability influences community composition, future research should explore how modulating these electron sinks may prevent pathogenic overgrowth and promote microbiome balance. Finally, the contribution of archaea to biofilm formation and electron transfer, well recognized in corrosion environments, remains underexplored in the gut. A deeper understanding of archaeal roles could provide new insights into community dynamics and health outcomes.

Bridging insights from corrosion-related EAM to the gut microbiome, particularly in the areas of biofilm formation, redox modulation, and interspecies interactions, holds significant potential for advancing microbiome engineering strategies. Understanding how environmental factors influence microbial behavior in one system can offer innovative approaches to manipulate and optimize microbial communities in others as well.

## Supplementary Material

Supplementary_material_v2_wraf112(1)

## Data Availability

All data generated or analysed during this study are included in this published article (and its supplementary information files).
