# Supplementary material for: Electroactive ecosystem insights from corrosion microbiomes inform gut microbiome modulation
Source: ISME J. 2025 May 31;19(1):wraf112. doi: 10.1093/ismejo/wraf112 (PMC12203069; doi:10.1093/ismejo/wraf112)
Supplement: Supplementary_material_v2_wraf112(1) [file supplementary_material_v2_wraf112(1).docx]

Electroactive ecosystem insights from corrosion microbiomes inform gut microbiome modulation

Authors:

Liam M. Jones^1*^, Sahar El Aidy^1,2^*

^1^ Department of Microbiome Engineering, Swammerdam Institute for Life Sciences, University of Amsterdam, Science Park 904, 1098 XH Amsterdam, Amsterdam, The Netherlands

^2^ Amsterdam Microbiome Expert Centre (AMEC), University of Amsterdam, Science Park 904, 1098 XH Amsterdam, Amsterdam, The Netherlands.

***Corresponding Author**

Liam M. Jones, Department of Microbiome Engineering, Swammerdam Institute for Life Sciences, University of Amsterdam, Science Park 904, 1098 XH Amsterdam, The Netherlands. E-mail: ✉ l.m.jones@uva.nl; Sahar El Aidy, Department of Microbiome Engineering, Swammerdam Institute for Life Sciences, University of Amsterdam, Science Park 904, 1098 XH Amsterdam, The Netherlands. E-mail: ✉ s.elaidy@uva.nl

**Running title:** Electroactive Microbiomes

# Supplementary material

**Table 1.** Corrosion-associated electroactive microorganisms included in the phylogenetic tree **(Fig 2)**.

| Microorganism | EET Mechanism | Genome Accension number | Reference |
| --- | --- | --- | --- |
| *Bacillus licheniformis* | MET | GCF_034478925.1_ASM3447892v1_genomic | [79] |
| *Desulfovibrio vulgaris* | DET | GCF_000015485.1_ASM1548v1_genomic | [95] |
| *Desulfuromonas acetoxidans* | DET | GCF_900111775.1_ASM90011177v1_genomic | [62] |
| *Desulfovibrio ferrophilus* | DET | GCF_003966735.1_ASM396673v1_genomic | [94] |
| *Electrothrix aarhusiensis* | DET | GCA_004028505.1_ASM402850v1_genomic | [76] |
| *Geobacter metallireducens* | DET | GCF_000012925.1_ASM1292v1_genomic | [28] |
| *Geobacter sulfurreducens* | DET | GCF_000007985.2_ASM798v2_genomic | [78], [90] |
| *Marinobacter atlanticus* | DET | GCF_000284615.1_ASM28461v1_genomic | [16] |
| *Methanococcus maripaludis* | DIET | GCF_002945325.1_ASM294532v1_genomic | [29] |
| *Methanosarcina barkeri* | DIET | GCF_000970025.1_ASM97002v1_genomic | [29] |
| *Methanothrix harundinacea* | DIET | GCF_000235565.1_ASM23556v1_genomic | [29] |
| *Pseudomonas aeruginosa* | DET | GCF_000006765.1_ASM676v1_genomic | [54] |
| *Shewanella oneidensis* | DET/MET | GCF_000146165.2_ASM14616v2_genomic | [24] |
| *Shewanlla putrefaciens* | DET/MET | GCF_016406325.1_ASM1640632v1_genomic | [80], [88], [89] |
| *Thermincola potens* | DET | GCF_000092945.1_ASM9294v1_genomic | [86] |

**Table 2.** Gut-associated electroactive microorganisms included in the phylogenetic tree **(Fig 2)**.

| Microorganism | EET Mechanism | Genome Accension number | Reference |
| --- | --- | --- | --- |
| *Akkermansia muciniphila* | DET | GCF_017504145.1_ASM1750414v1_genomic | [72], [73], [93] |
| *Bacteroides fragilis* | MET | GCF_000025985.1_ASM2598v1_genomic | [75] |
| *Bifidobacterium adolescentis* | MET | GCF_000010425.1_ASM1042v1_genomic | [67] |
| *Blautia obeum* | MET | GCF_025147765.1_ASM2514776v1_genomic | [40] |
| *Clostridium cochlearium* | Unknown | GCF_900187165.1_52121_G02_genomic | [56] |
| *Desulfovibrio desulfuricans* | DET | GCF_017815575.1_ASM1781557v1_genomic | [59] |
| *Enterobacter cloacae* | DET/MET | GCF_905331265.2_AI2999v1_cpp_genomic | [101] |
| *Enterococcus faecalis* | MET | GCF_000393015.1_Ente_faec_T5_V1_genomic | [83] |
| *Escherichia coli* | MET | GCF_000008865.2_ASM886v2_genomic | [81] |
| *Faecalibacterium prausnitzii* | MET | GCF_000154385.1_ASM15438v1_genomic | [13], [92] |
| *Klebsiella pneumoniae* | DET | GCF_000240185.1_ASM24018v2_genomic | [96] |
| *Limosilactobacillus reuteri* | MET | GCF_003703885.1_ASM370388v1_genomic | [58] |
| *Roseburia intestinalis* | MET | GCF_900537995.1_Roseburia_intestinalis_strain_L1-82_genomic | [102] |
| *Ruminococcus intestinalis* | MET | GCF_014288065.1_ASM1428806v1_genomic | [40] |
| *Streptococcus agalactiae* | DET | GCF_001552035.1_ASM155203v1_genomic | [58] |
| *Veillonella parvula* | MET | GCF_900186885.1_48903_D01_genomic | [103] |
